# The Effect of Temperature on the Stability of African Swine Fever Virus BA71V Isolate in Environmental Water Samples

**DOI:** 10.3390/pathogens12081022

**Published:** 2023-08-08

**Authors:** Eleni-Anna Loundras, Christopher L. Netherton, John Flannery, Michael J. Bowes, Linda Dixon, Carrie Batten

**Affiliations:** 1 The Pirbright Institute, Ash Road, Pirbright, Woking GU24 0NF, UK; eleni-anna.loundras@pirbright.ac.uk (E.-A.L.); christopher.netherton@pirbright.ac.uk (C.L.N.); johnt.flannery@tus.ie (J.F.); linda.dixon@pirbright.ac.uk (L.D.); 2 Department of Animal Health, Technological University of the Shannon, Athlone Campus, N37HD68 Athlone, Ireland; 3 Centre for Ecology and Hydrology, Wallingford Oxon OX10 8BB, UK; mibo@ceh.ac.uk

**Keywords:** African swine fever virus, stability, infectivity, environmental water, groundwater

## Abstract

African swine fever virus (ASFV) is known to be very stable and can remain infectious over long periods of time especially at low temperatures and within different matrices, particularly those containing animal-derived organic material. However, there are some gaps in our knowledge pertaining to the survivability and infectivity of ASFV in groundwater. This study aims to determine the stability and infectivity of the cell culture-adapted ASFV strain BA71V by plaque assay after incubation of the virus within river water samples at three different environmentally relevant temperatures (4 °C, 15 °C, and 21 °C) over the course of 42 days. The results from this study indicate that ASFV can remain stable and infectious when maintained at 4 °C in river water for more than 42 days, but as incubation temperatures are increased, the stability is reduced, and the virus is no longer able to form plaques after 28 days and 14 days, respectively, when stored at 15 °C and 21 °C. Characterizing the survivability of ASFV in groundwater can allow us to develop more appropriate inactivation and disinfection methods to support disease control and mitigate ASFV outbreaks.

## 1. Introduction

African swine fever virus (ASFV), the causative agent of African swine fever (ASF), a viral hemorrhagic disease with case fatality rates in domestic pigs and wild boars, is the only member of the *Asfaviridae* family [1,2]. Although highly virulent strains of the virus can cause very high fatality rates close to 100%, less virulent strains have been described which cause reduced fatality. ASFV is known to persistently infect warthogs, bushpigs, and soft ticks of the genus *Ornithodoros,* which act as a vector for viral transmission [3,4]. Since the 2007 incursion of ASFV in Georgia, the virus has been rapidly spreading through eastern and mainland Europe [5]. In mid-2018, ASFV entered China and has subsequently spread throughout Southeast Asia [6,7]. The growing presence of the virus has had a subsequent economic impact, particularly on the global pork production industry as a result of millions of infected pigs being destroyed. This highlights the increasing demand for an inexpensive and simple method of disinfection to remove infectious ASFV from surfaces, silage, and other waste from pig rearing facilities since currently no commercial vaccines or antiviral treatments are available. Vector control and preventative measures such as strict quarantine and biosecurity, restrictions on animal movement, and the slaughter of affected animals are the main strategies for disease control and the primary options for mitigating ASFV outbreaks [8]. 

ASFV is known to be very stable and can remain infectious over a long period of time, particularly when stored at temperatures below 4 °C. However, some gaps in our knowledge remain regarding ASFV survival in different matrices including feed, fomites, and silage. There have been a number of studies detailing the survival of ASFV in animal feed and bedding, as well is in infected carcasses and material of pig origin, and within the underlying soil under various environmental conditions [9,10,11,12,13,14,15,16]. However, the ability of ASFV to survive in groundwater has not yet been exclusively studied. A recent publication [17] has speculated at a correlation between the frequency in infected animals and their proximity to bodies of water, but no further investigations were made. This gap in the knowledge of ASFV survivability and transmissibility in groundwater urgently needs to be addressed. Controlled laboratory experiments have found that an infectious dose for ASFV in liquid is possible and may be very low when compared to that found in feed (10^0^ TCID_50_ compared to 10^4^ TCID_50_ in feed) [18], which points to a requirement to clarify the survivability of ASFV in environmentally relevant liquid matrices such as groundwater. Furthermore, the ability to characterize the survival time of the virus in groundwater will allow for the development of more appropriate inactivation and disinfection methods that can then be implemented to support disease control.

**Figure 1:**
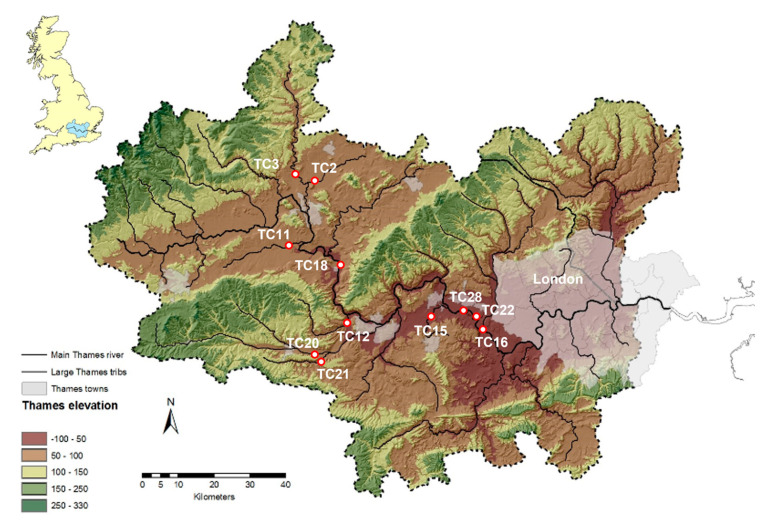
Map of the Thames catchment showing the location of the sampling sites of each of the 11 samples provided by UKCEH and the associated sample numbers used in this study. Locations of the sampling sites are located along the main River Thames and several tributaries.

In the study outlined in this brief report, we use an ASFV genotype I cell culture-adapted isolate BA71V [19,20,21] to inoculate different water samples collected from various locations along the River Thames and its major tributaries (Figure 1). Environmental river water samples with a defined chemical composition were provided by the UK Centre for Ecology & Hydrology (UKCEH) for these experiments [22] (Supplementary Data). River water was chosen as the environmentally relevant matrix for the incubations as it was considered the most appropriate to determine ASFV survival since it contains the biological and physicochemical parameters which can contribute to the inactivation of viruses in the environment when compared to laboratory-grade distilled water. Furthermore, untreated water from natural courses is considered the most likely source of contamination to native pigs rather than municipal wastewaters. 

## 2. Materials and Methods

### 2.1. Propagation of BA71V Virus Stocks

Briefly, Vero cells (African green monkey cells, ECACC 84113001) maintained in Dulbecco’s modified Eagle’s medium (DMEM) (Sigma-Aldrich, Burlington, MA, United States) supplemented with 10 % fetal bovine serum (FBS) (Gibco, Waltham, MA, USA) were infected with 1 ml of cell culture-adapted ASFV strain of BA71V crude stock and left to propagate for 72 h or until 100% cytopathic effect was observed [20,23]. Crude cell lysates and supernatant from infected cells were harvested and clarified by centrifugation at 1000× RPM for 10 min. Cell pellets were processed by freezing at −70 °C and thawing at 37 °C for three cycles and subsequently sonicated for 10 s for three cycles at 50% amplitude to enable the release of any virus within the cell debris. Cell debris was removed by centrifugation and the supernatants were combined, aliquoted, and stored at −70 °C.

### 2.2. Sampling of Environmental Water

Water samples were collected from across the Thames catchment. Samples were collected at four sites along the River Thames and from a further 7 tributaries on the Rivers Kennet, Enborne, Ray, Cherwell, Pang, The Cut, and Ock by UKCEH as part of their Thames Initiative research platform [22]. General water quality data (water temperature, dissolved organic carbon, pH, alkalinity, and the concentrations of phosphorus and nitrogen species, chlorophyll, and suspended sediments) were provided (Supplementary Data: Table S1). 

### 2.3. Titration of Virus Stocks

ASFV BA71V titer was determined by virus titration by ten-fold dilution prior to adsorbing onto confluent Vero cell monolayers in 6-well plates. The cells were incubated in 5 % CO_2_ at 37 °C for 1 h prior to being overlaid with 1.375% Eagle’s media overlay (produced in-house). The overlay was supplemented with 4% FBS (Gibco) 1% Avicel (RC-591 MCC/Carboxymethylcellulose sodium) solution (Dupont), 0.1 % penicillin/streptomycin (Sigma-Aldrich), and 0.1 % L-glutamine (Gibco). Overlay reagents were combined and incubated at 42 °C while the cells were adsorbing the virus [23]. The cells were then incubated for 6 days to allow for plaque formation to occur and stained with crystal violet solution (Alfa Aesar, Haverhill, MA, USA) [23]. A minimum titer of 1 × 10^4.5^ plaque-forming units (p.f.u) per ml was required for this study in order to identify a 4 −log^10^ reduction in infective titer [24].

### 2.4. Water Inactivation

ASFV BA71V strain stock virus propagated in Vero cells as described above was diluted 1:10 in 900 µL water samples collected from the Thames Valley tributaries. These were stored at 4 °C, 15 °C, and 21 °C (± 2 °C) for 0, 7, 14, 21, 28, and 42 days. Following incubation, the titer of the virus was determined by a plaque assay as described previously [23]. Controls used stock virus diluted in 900 µL laboratory-grade distilled water (dH_2_O) supplemented with 1% FBS (Gibco). These were also incubated for the same length of time at the appropriate temperatures.

## 3. Results

**Figure 2:**
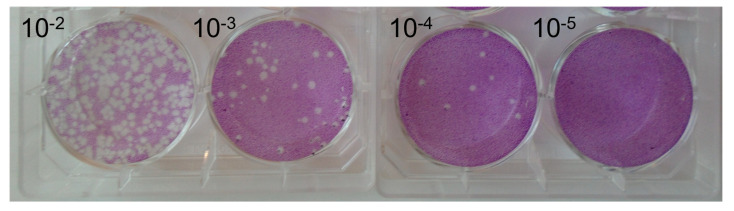
Wells of a 6-well plate showing a ten-fold dilution of ASFV BA71V from 10^−2^ to 10^−5^. Virus titer is calculated by counting the number of plaques formed. This is a representative image of the plaque assay. Morphology and size of individual plaques were not deemed different between virus incubated in environmental water samples and control distilled water samples.

ASFV BA71V was incubated in river water from eleven different locations (TC2-28; Table 1) along the Thames Valley (Figure 1) at three environmentally relevant temperatures (4 °C, 15 °C, and 21 ± 2 °C) for 42 days. Samples of incubated virus were taken after 0, 7, 14, 21, and 42 days and the titer, and subsequent infectivity, of the virus was determined by plaque assay on Vero cells. A 4 −log^10^ reduction in titer, calculated from the formation of the number of p.f.u per ml, was required to consider a particular sample no longer infectious in cell culture [24]. Figure 2 depicts a representative plaque assay showing a reduction in the number of plaques based on a ten-fold dilution series. It is important to note that the morphology of the plaques was not changed by the matrix in which the virus was incubated.

**Figure 3:**
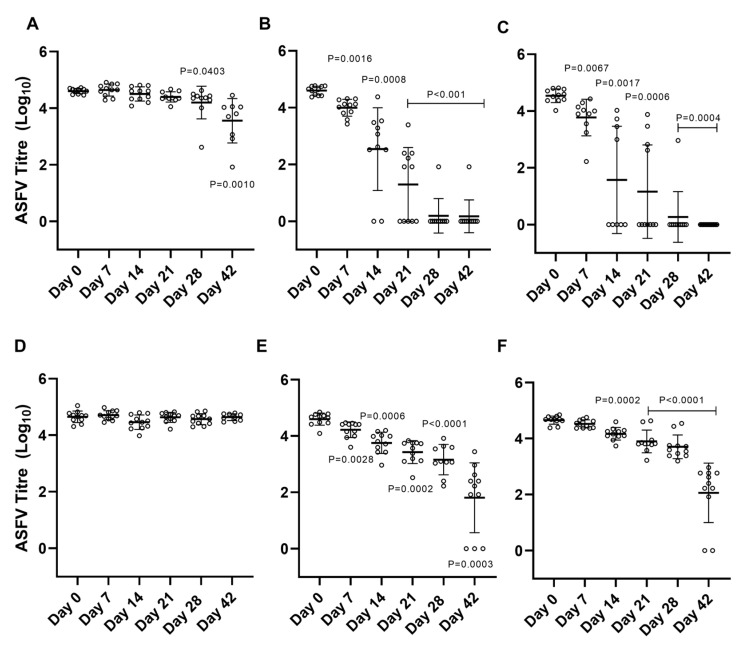
Average ASFV BA71V titer (log^10^ p.f.u) over time when incubated in environmental water samples at 4 °C (**A**), 15 °C (**B**), and 21 °C (**C**), and average ASFV BA71V titer over time incubated in control water samples containing 1% FBS at 4 °C (**D**), 15 °C (**E**), and 21 °C (**F**). Statistical analyses (mixed-effect analysis and one-way ANOVA) using both Dunnett’s and Tukey’s multiple comparison tests undertaken using GraphPad Prism 8 software. Individual *p*-values defined on graph.

The results generated from this assay show that ASFV BA71V maintains its stability and infectivity in all river water samples when incubated at 4 °C for more than 42 days (Figure 3A). The results show that there is an average 1.05 −log^10^ (±0.41) reduction in virus titer over the 42-day incubation period at 4 °C. This reduction in virus titer, although statistically significant when compared to the titers of virus incubated in environmental water samples for 0 days, is not even reduced by 1 −log^10^ and is therefore not considered significant in the context of the stability and infectivity of the virus isolate in cell culture. However, as the incubation temperature is increased, the stability, and therefore infectivity, of ASFV BA71V was reduced significantly both in the context of statistical analyses and in the context of the reduction in virus titer. The average virus titer was reduced by 4.41 −log^10^ (±1.83) and 4.27 −log^10^ (±1.81), respectively, after 28 days at 15 °C (Figure 3B) and also after 28 days at 21 °C (Figure 3C). With the exception of a couple of individual samples, ASFV stability and subsequent infectivity was reduced to only 14 days when incubated at 21 °C; however, the average reduction in virus titer was only 2.96 −log^10^ (±1.54). Comparatively, ASFV BA71V incubated in the control distilled water sample (dH_2_O + 1 % FBS) maintained its infectivity with a minimal average reduction in viral titer (0.02 −log^10^) at 4 °C (Figure 3D) and an average 2.69 −log^10^ (±0.96) reduction in infectivity by 42 days of incubation at both 15 °C and 21 °C (Figure 3E,F). 

These data also highlight the natural variability observed within the river water samples and the effect this has on virus stability and subsequent infectivity in cell culture. ASFV BA71V, when incubated in the environmental water samples, appears to have greater within-sample titer variability than when it is incubated in control distilled water samples which only contain 1 % supplemental FBS, as evidenced by the narrower margins of error within Figure 3D–F when compared to Figure 3A–C.

**Figure 4:**
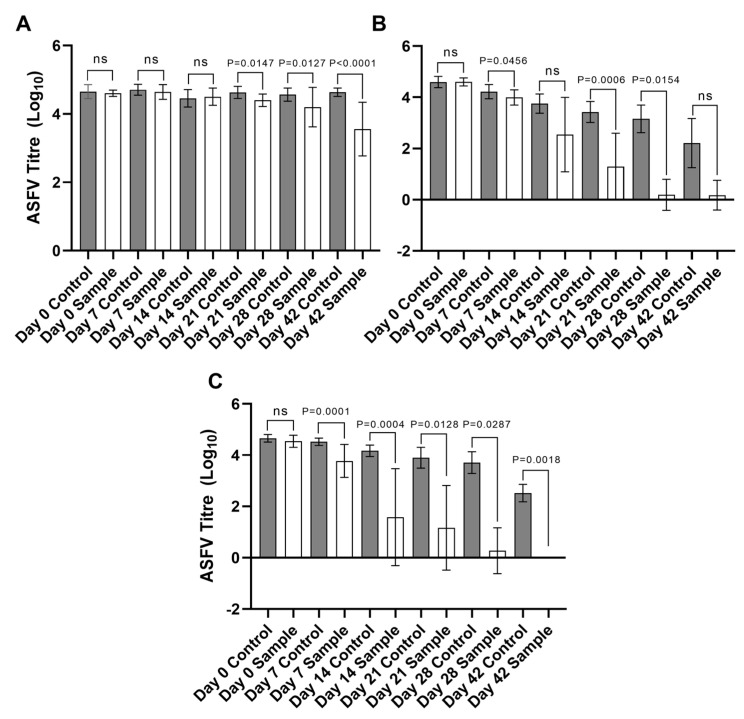
Comparison of the average ASFV BA71V titer (log^10^ p.f.u) over time when incubated in environmental water samples and control distilled water samples containing 1% FBS at 4 °C (**A**), 15 °C (**B**), and 21 °C (**C**). Statistical analyses (Paired *t*-Test) undertaken using GraphPad Prism 8 software. Individual *p*-values defined on graph, not statistically significant (ns).

The difference in observed viral titers when comparing virus stability and infectivity from ASFV BA71V incubated in environmental water samples to virus that was incubated in the control distilled water samples was further investigated (Figure 4A–C). When comparing average ASFV BA71V titers across all eleven environmental water samples of those incubated at 4 °C, the differences in titers were not statistically significant up to 21 days (Figure 4A). As incubation times increase and as the temperatures increase, the stability and infectivity of ASFV BA71V are shown to reduce. The reduction in virus titers is more pronounced in the environmental samples and as the temperatures are increased to 15 °C and 21 °C when compared to those incubated in control distilled water samples (Figure 4B,C). The presence of serum as 1 % FBS in the control distilled water samples may be contributing to the stability of the virus within the matrix.

## 4. Discussion

The results from this preliminary study confirmed the relative stability of ASFV BA71V within river water, a previously unstudied environmental matrix. We also demonstrated the ability for the virus to persist and maintain infectivity over time and at different environmentally relevant temperatures. The results are generally in agreement with previously published data [13,14,15] showing the stability and infectivity of ASFV when stored in a variety of different matrices and at different temperatures. Our results show that, on average, ASFV BA71V was able to remain infectious after being stored for 42 days at 4 °C, but as the temperature of the environmental water samples increased to 15 °C and 21 °C, the stability of the virus was reduced to 28 and 14 days, respectively. A 4 −log^10^ reduction in titer was not seen until virus was incubated for 28 days at both 15 °C and 21 °C. The mean temperature of the river water in the three weeks prior to sampling (18 October 2021) was recorded as 16.4 °C, with the lowest temperature recorded as 13.5 °C and the highest at 18.1 °C (Supplementary Data). It is expected that the water temperature fluctuates seasonally; however, it should remain within the temperature thresholds tested in this study (4 °C, 15 °C, 21 ± 2 °C). This preliminary discrete study provides us with a functional window that assesses the risk in ASFV survivability in groundwater in the UK. 

For this study, water was sampled from several locations within the Thames catchment area in order to allow for any preliminary identification of any potential physicochemical properties that may have affected virus stability, for example, pH. The large number of river water samples being tested allowed for increased natural variability, which can be seen in individual sample data points in Figure 3. An example of this variability can be seen in titers calculated from ASFV BA71V incubated in Sample 6 river water (TC28) at 4 °C for 28 and 42 days. These data points trended towards a lower viral titer; however, the viral titers calculated from the same sample at 21 days post-infection when incubated at 21 °C trended high in comparison to other sample data points from ASFV BA71V incubated in other river water samples. It is important to note that the variability seen within samples is not consistent across time points or temperatures, indicating that the chemical properties of the environmental water samples as supplied to us by the UKCEH did not appear to have a significant effect on virus stability or infectivity. However, further studies to address changes and differences in the physicochemical compositions of the groundwater, particularly during different seasons, and the effect those might have on ASFV stability and infectivity over time, still need to be undertaken.

Interestingly, the addition of protein in the form of serum (1 % FBS) within our control distilled water samples improved the stability of ASFV BA71V both over time and at increasing temperatures when compared to the virus incubated within our environmental water samples (as detailed in Figure 3 and Figure 4). The presence of animal-derived protein could be a contributing factor to ASFV being able to persist for long periods of time within blood and other animal-derived products. This has been evidenced in previous studies when determining the stability and survivability of ASFV within meat products and infected animal carcasses [13,14,15]. The presence of these animal-derived products, such as serum, could be a factor to consider when assessing the stability of the virus if it were found within infected animal blood and bodily fluids, or in carcasses of infected animals that have been discarded in water courses such as lakes and rivers, particularly as the majority of ASFV would be associated with red blood cells. 

These results can help to dictate the timescales for quarantine and exclusion zones in regions of localized ASFV outbreaks and can also inform us on the effects of temperature and climate change on the persistence and spread of ASFV in the environment. The results highlight the risk of the spread of the virus into groundwater from infected carcasses or persistently infected animals. Two recent studies point to changes in the climate that are favorably influencing the spread of ASFV due to the effects on vector–host distribution and relationship, and due to changes in local environments such as in average annual rainfall and changes in seasonal temperatures [17,25]. As such, it is prudent to understand the ability for ASFV to persist in the natural environment in order to become better informed in our attempts to control and mitigate the spread of the disease.

## Figures and Tables

**Table 1:** Table summarizing sampling location codes with associated sample number assigned during experimentation.

Sampling Location Code	Sample Number
TC2	S8
TC3	S10
TC11	S2
TC12	S4
TC15	S3
TC16	S9
TC18	S11
TC20	S1
TC21	S7
TC22	S5
TC28	S6

## Data Availability

Data are contained within the article or Supplementary Material.

## Supplementary Materials

The following supporting information can be downloaded at: https://www.mdpi.com/article/10.3390/pathogens12081022/s1; Table S1: Thames Water Quality Data.
